# Grief and Posttraumatic Growth Among Chinese Bereaved Parents Who Lost Their Only Child: The Moderating Role of Interpersonal Loss

**DOI:** 10.3389/fpsyg.2020.558313

**Published:** 2020-10-09

**Authors:** Xin Xu, Jun Wen, Ningning Zhou, Guangyuan Shi, Renzhihui Tang, Jianping Wang, Natalia A. Skritskaya

**Affiliations:** ^1^Beijing Key Laboratory of Applied Experimental Psychology, National Demonstration Center for Experimental Psychology Education, Faculty of Psychology, Beijing Normal University, Beijing, China; ^2^Center for Complicated Grief, Columbia School of Social Work, New York, NY, United States

**Keywords:** shidu parents, grief, posttraumatic growth, moderating effect, interpersonal loss, socio-economic loss

## Abstract

**Objective**: Losing the only child is considered as the most severe kind of bereavement. It can trigger intense grief symptoms along with loss of psychosocial resources, but meanwhile, it can also lead to posttraumatic growth (PTG). The current study aimed to examine (a) whether a curvilinear relationship exists between grief and PTG and (b) the moderating role of resources-loss among Chinese bereaved parents who lost their only child (shidu parents).

**Methods**: One hundred and ninety-nine shidu parents from five provinces completed the assessment packet covering prolonged grief symptoms and PTG, as well as resource loss. Hierarchical regression analyses were computed to examine the curvilinear relationship and the moderating effect of interpersonal loss and socio-economic loss.

**Results**: There was no significant curvilinear relationship between grief and PTG in shidu parents. Under the high levels of interpersonal loss, shidu parents’ PTG scores decreased as the severity of grief increased. However, the socio-economic loss did not show a significant moderating effect.

**Conclusion**: The results of the current study did not show a significant curvilinear relationship between grief and PTG in shidu parents. High levels of grief coupled with high levels of interpersonal loss may interfere with their ability to achieve PTG. Therefore, evaluating degrees of interpersonal loss seems to be an important part of the treatment process when working with shidu parents. To facilitate their adaptation from a positive perspective, helping them maintain existing or develop new interpersonal relationships may be helpful.

## Introduction

As an old Chinese saying states, “there are three biggest misfortunes in life: fatherless in youth, widowed in middle age, and childless in old age,” loss of a child is considered the most severe kind of bereavement (Zetumer et al., 2015). It often leads to intense emotional pain and a higher rate of prolonged grief disorder (PGD; Kersting et al., 2011; Newson et al., 2011; Zhou et al., 2018). According to the 11th edition of the International Classification of Diseases (ICD-11; World Health Organization, 2018), PGD is characterized by longing for or persistent preoccupation with the deceased accompanied by intense emotional pain (e.g., sadness, guilt, and anger), as well as functional impairment, which persist for more than 6 months. Bereaved parents often appear to hold certain maladaptive cognitions about death (Skritskaya et al., 2017; Kokou-Kpolou et al., 2018). Bereaved parents with PGD demonstrated more intense grief symptoms, self-blame, and suicidality than other bereaved people with PGD (Zetumer et al., 2015).

What is more bitter than the death of a child is losing the only child in a context where it is a cultural imperative to continue family bloodlines, and children are the source of care for aging parents (Zheng and Lawson, 2015). Because of the previous “One-Child Policy” strictly implemented in China from 1980, there is a special kind of bereaved parents called shidu parents. In the policy targeted at this population, they are defined as bereaved parents over the age of 49 who have lost their only child, and they can receive a monthly subsidy from the government (Wang and Hu, 2019; Zhou et al., 2020). Chinese culture attaches great importance to filial piety and the continuation of family lineage. As reflected by the Confucian saying “having no posterity is extremely nonfilial,” it is a cultural imperative to have a child (Pan et al., 2016). Therefore, shidu parents are not only afflicted by child loss but also suffer from identity loss and social stigmatization (Zheng and Lawson, 2015). Compared to Chinese parents with a living child or who had given birth again, they had a higher risk of intense grief, depression, anxiety, posttraumatic stress, and chronic diseases (Xu et al., 2014; Zheng et al., 2017; Cao et al., 2018; Yin et al., 2018). Additionally, compared with a prevalence of PGD of about 10% in general bereaved people (Lundorff et al., 2017), the prevalence rate in shidu parents was 35.5% (Zhou et al., 2020). Although grief intensity may vary over time, grief would not go away entirely throughout shidu parents’ whole life (Wei et al., 2016; Simon et al., 2020).

Alarmingly, the number of shidu parents was more than 1.5 million in 2015 in China, and the number of shidu families is expected to reach about 4.5 million by 2050 (Wang, 2016). It is imperative to learn more about their bereavement reactions and then design targeted clinical interventions for them. Despite grief being a universal experience and some valuable findings regarding bereaved parents have been discussed, grief symptoms and expression vary by culture (Xiu et al., 2016; Kokou-Kpolou et al., 2020; Stelzer et al., 2020). Given that shidu parents are affected by Chinese familism and Confucianism culture (Zhang and Jia, 2018; Shi et al., 2019), there may be some unique characteristics among shidu parents.

Another concern about shidu parents is that the only child’s death usually will lead to secondary resources loss. Interpersonal and socio-economic loss may be the most common loss they have experienced. In Chinese culture, some people hold an opinion that the death of children is a result of karma (Chan et al., 2005; Zheng et al., 2017). Death is a punishment for previous immoral behavior. Usually, the death of a child is a taboo topic in China. Even nowadays, family members rarely talk about the deceased openly (Cacciatore and DeFrain, 2015). Therefore, shidu parents usually experienced a form of disenfranchised grief in China. This social and cultural context may make it more difficult for shidu parents to cope with child-loss. Shidu parents would deliberately or passively end up in social isolation because of their child-death (Wei et al., 2016). Some of them even quit their job, sell their house, and move to a different place. Therefore, the loss of the only child often results in interpersonal loss. Additionally, because of Chinese traditional filial piety culture, adult children are the main care providers, giving emotional and financial support to their parents (Zheng et al., 2017; Cao et al., 2018). Therefore, losing the only child also leads to economic losses, especially in rural areas (Liang et al., 2019). Undoubtedly, these resources loss may, in turn, affect shidu parents’ psychological status (Lambert et al., 2018).

Although the negative consequences of child-loss have been well-documented, studies reported that people who struggle with traumas could also experience positive psychological changes, such as posttraumatic growth (PTG; Tedeschi and Calhoun, 2004). Therefore, it is not surprising that bereaved parents may achieve certain levels of PTG (Moore et al., 2015; Albuquerque et al., 2018; Waugh et al., 2018), even after losing the only child (Pan et al., 2016; Zhou et al., 2018). Three facets of growth among Chinese shidu parents have been identified in a previous study (Xu et al., under review): (1) A changed philosophy of life. For example, survivors will re-evaluate what is important in life. (2) Changes in self. Shidu parents may develop new interests or establish new paths for their lives, instead of mainly focusing on their children as they did in the past. Shidu parents may feel that they can handle anything after going through such a painful loss (Tedeschi and Calhoun, 2004). (3) Changes in relationships with others. Shidu parents may become more compassionate, particularly toward those who are in a similar situation (Calhoun et al., 2010). PTG is worthy of being investigated in trauma research and integrated into clinical practice (Zoellner and Maercker, 2006). Although PTG is a universal phenomenon, its manifestation may have cross-cultural differences (McDiarmid and Taku, 2016). Considering culture impacts people’s reflection after trauma (Kashyap and Hussain, 2018), more studies about PTG among shidu parents are highly needed, which may help therapists design interventions from a positive perspective (Roepke, 2015).

It is worth noting that grief and PTG can, and do, coexist after bereavement (Tedeschi et al., 2007; Zhou et al., 2018). Lots of studies have examined the relationship between grief and PTG but with conflicting results. Some studies show a negative correlation (Engelkemeyer and Marwit, 2008), some positive (Xu et al., 2015), or no significant correlation at all (Salloum et al., 2019). These inconsistent findings suggest that the relationship between grief and PTG may vary in different bereaved samples. Based on a model of growth in grief, some degree of emotional distress may urge bereaved people to manage the pain, find ways to understand death, and finally accept the changed world. Too low levels of grief may be not enough to challenge their assumptive world beliefs and facilitate them to change (Calhoun et al., 2010). However, severe grief may exhaust people’s resources to achieve growth (Tedeschi and Calhoun, 1995). Therefore, some researchers suggested that certain grief levels may be useful for producing PTG, and an inverted U-shape relationship might be adequate for grief and PTG (Currier et al., 2012; Yilmaz and Zara, 2016; Eisma et al., 2019). Considering that both grief and PTG may be affected by culture and society (Cormio et al., 2015), simply copying a Western model to another culture is inadvisable (Neimeyer, 2012). Chinese bereaved parents are in a collectivist and family-centered culture, while European and American parents are affected by individualism (Xiu et al., 2016). Whether the curvilinear relationship between grief and PTG exists among shidu parents is still worth exploring.

The impact of different moderators affecting distress and PTG can also partly explain the ambiguous relationship between grief and PTG (Shiri et al., 2008; Tian and Solomon, 2018). We speculated that resource loss might play a moderating role between grief and PTG, because low-to-moderate resources loss may motivate people to use active coping and instrumental coping to deal with loss and grief (Hobfoll, 1991). Successful coping with more grief can provide the potential for greater PTG (Calhoun et al., 2010; Waugh et al., 2018). However, if resources losses are severe and individual’s resources are exhausted, they may be defensive, aggressive, and even irrational (Hobfoll et al., 2018). In this situation, people with very few resources may be overwhelmed and too preoccupied with their difficulties. Serious maladjustment may ensue following the stressful experience (Tedeschi and Calhoun, 2004). It is hard for bereaved people to deal with the intense grief to achieve personal growth (Currier et al., 2012). Therefore, the relationship between grief and PTG might differ for individuals with different levels of resource loss.

### The Current Study

The present study aimed to examine (a) whether a curvilinear relationship exists between grief and PTG among shidu parents and (b) whether resources-loss affects the relationship of grief and PTG. Two hypotheses are as follows: (1) based on the evidence from previous studies (Currier et al., 2012; Yilmaz and Zara, 2016), there is an inverted U-shape model between grief and growth in shidu parents; (2) the relationship between grief and PTG is different under different levels of resources-loss. Under low-to-moderate levels of resources loss, shidu parents with higher levels of grief report higher PTG. When under high levels of resources loss, shidu parents’ intense grief may be related to lower PTG.

## Materials and Methods

### Procedures

The data used in the current study were a part of a larger project “Constructing a psychological help system for Chinese shidu parents, based on a popular-based survey” collected from April 2017 to August 2017 across five provinces. The project was approved by the Ethics Committee of Beijing Normal University. With the help of the community institutions and public interest organizations (e.g., self-help groups for shidu parents), we recruited shidu parents by a convenient sampling method. For a detailed recruitment description, see Zhou et al. (2020).

### Participants

A total of 211 shidu parents completed the questionnaire packet. Seven questionnaires were excluded because missing items were more than 20% of the data. Five participants who lost their child within 6 months were also excluded from the analyses, because they may be in the period of acute grief (Shear, 2015; World Health Organization, 2018). The validation rate for the questionnaires of the present study was 94.3%. The final sample size was 199, including 71 males and 128 females. The participants came from five provinces: Anhui (*n* = 21), Jilin (*n* = 52), Henan (*n* = 80), Jiangsu (*n* = 7), and Hunan (*n* = 39). Their average age was 60.92 (*SD* = 6.98) years, with the average length of loss was 8.30 (*SD* = 6.00) years. More detailed descriptive information of the sample is presented in Table 1.

**Table 1:** Demographic characteristics of participants.

Demographic variables	*M* (SD) or *N* (%)	Loss-related variables	*M* (SD) or *N* (%)
Gender		Gender of the child	
Male	71 (35.7%)	Male	152 (76.4%)
Female	128 (64.3%)	Female	46 (23.1%)
Missing	0 (0.0%)	Missing	1 (0.5%)
Age	60.92 (6.98)	Age of the child	25.18 (8.73)
Missing	1 (0.5%)	Missing	11 (5.5%)
Education level		Time since loss (years)	8.30 (6.00)
Primary school	44 (22.1%)	Missing	20 (10.1%)
Middle/high school	134 (67.3%)	Cause of death	
College or above	15 (7.5%)	Non-violent	101 (50.8%)
Missing	6 (3.0%)	Violent	98 (49.2%)
Marriage status		Expectation of the death	
Married	57 (28.6%)	No	167 (83.9%)
Divorced/widowed	139 (69.8%)	Yes	31 (15.6%)
Missing	3 (1.5%)	Missing	1 (0.5%)
Month income (USD)	294.14 (195.39)	Having a grandchild not	
Missing	6 (3.0%)	No	138 (69.3%)
		Yes	61 (30.7%)

### Measurement

#### Sociodemographic Information

A brief demographic questionnaire was used to collect basic information regarding each participant’s gender, age, education, marital status, monthly income, as well as relevant information of the deceased children.

#### Prolonged Grief Symptoms

The severity of grief symptoms was measured by the Prolonged Grief Disorder Questionnaire (PG-13; Prigerson et al., 2009). Two items assess separation distress, and nine items are related to cognitive, emotional, and behavioral reactions. A five-point scoring system was used (1 = never and 5 = several times a day) to rate the first 11 items. The last two items regarding the length of bereavement and functional impairment were to be answered “yes” or “no.” Total scores of the 11 items excluding the functional impairment and length items were calculated to represent the severity levels of grief symptoms (Pohlkamp et al., 2018). The Chinese version of PG-13 was shown to have good validity and reliability and was widely used in bereaved people (He et al., 2014; Zhou et al., 2018). The Cronbach’s alpha of the 11-items was 0.910 in the current study.

#### Posttraumatic Growth

Posttraumatic growth inventory (PTGI) is the most widely used tool to assess positive psychological changes after trauma (Tedeschi and Calhoun, 1996). The Chinese version of PTGI was obtained by a back-translation method. It was translated into Chinese from the original version by two psychology doctoral students, and then translated back into English by a professional English postgraduate and finally revised by a clinical psychologist. A previous study found that the original five-factor model was not suitable for Chinese shidu parents (no convergent; Xu et al., under review). Through parallel analysis and principal components, a revised 17-item, three-factor structure version (Life philosophy, Self-changes, and Relationships with others) of PTGI for Chinese shidu parents (PTGI-CS) was deemed appropriate [root mean square error of approximation (RMSEA) = 0.058; comparative fit index (CFI) = 0.920; Tucker-Lewis index (TLI) = 0.905; and standardized root mean squared residual (SRMR) = 0.054]. According to the criteria used in previous studies (Tedeschi and Calhoun, 1996; Rodriguez-Rey et al., 2016), item 5, item 10, and item 13 in the original version were eliminated because of cross-loadings, and item 18 was eliminated because of factor loadings less than 0.50 (unpublished results). Items were rated on a six-point scale ranging from 1 (“I did not experience this change at all after losing the child”) to 6 (“I experienced this change greatly after losing the child”). Total scores ranged from 17 to 102, with higher scores reflecting greater growth. The Cronbach’s alpha of the PTGI-CS was 0.879 in the current study.

#### Resources Loss

Interpersonal loss and socio-economic loss were assessed by a six-item Resources Loss Scale (RLS). The former was measured by four items from the Conservation of Resources Evaluation (COR-E; Hobfoll and Lilly, 1993; Heath et al., 2012), including “Feeling that you are a person of great value to other people”; “Stability of your family”; “Intimacy with at least one friend”; and “Intimacy with at least one family member.” Two items related to the socio-economic loss were adapted from previous studies (Canetti et al., 2010), including “faith in government,” and “financial resources (e.g., money or property).” Participants were asked to use 0 (“no loss”), 1 (“a little loss”), 2 (“many losses”), and 3 (“great losses”) to indicate to what extent they have lost the following resources in the past year because of losing the only child. Higher scores are related to higher levels of resources-loss. The Chinese version of this measure was obtained by a back-translation method. The results of confirmatory factor analysis (CFA) were: RMSEA = 0.093, CFI = 0.954, TLI = 0.914, and SRMR = 0.034. The Cronbach’s α coefficients were 0.840 for the whole RLS, 0.823 for the interpersonal loss, and 0.566 for the socio-economic loss. Considering all the model fit indices and Cronbach’s α coefficients, this two-factor, six-item scale was applicable in the current study.

### Data Analyses

Harman’s single-factor test was used to determine whether a common method bias existed before analyzing the data. All observed variables (grief, PTG, and resource-loss) were included in an exploratory factorial analysis. Eight factors’ eigenvalues were greater than 1, and the first of which explained 22.25% of the total variance of the data. The first factor explained far less than 50% of the variance (Aguirre-Urreta and Hu, 2019). These results suggested that the possibility of common method bias was withdrawn, and further analysis could be conducted. Given the maximum percentage of missing values was 3.0% for RLS item 4, missing values of questionnaire items were imputed using the Expectation-Maximization algorithm. Then CFA was conducted to confirm the structure validity of the PTGI-CS and the Resources Loss Scale. The values of SRMR < 0.08, RMSEA < 0.08, CFI > 0.90, and TLI > 0.90 indicate an adequate or good model fit (Westland, 2015).

Descriptive statistics were used to summarize descriptive information of participants’ demographic characteristics and psychosocial variables. Pearson correlations analyses were carried out to examine the relationships between socio-demographic variables and key research variables. Next, following the procedure suggested by Aiken and West (1991), for scores of PTGI-CS as outcomes, series hierarchical multiple regression (HMR) analyses were conducted to test our two hypotheses. Model 1 was used to explore the moderating role of interpersonal loss, while Model 2 for socio-economic loss. A systematic review of bereaved parents’ PTG suggested that gender, time since the death, and cause of death may impact PTG (Waugh et al., 2018), and the continuation of family lineage is important in China. Therefore, the gender of participants (0 = male and 1 = female), time since the loss (years), cause of death (0 = non-violent, such as chronic disease; 1 = violent, such as an accident), and having a grandchild or not (0 = no and 1 = yes) were entered as covariates in step 1; grief was entered in step 2; squared grief levels (grief^2^) was entered in step 3 to assess the curvilinear relationship between grief and PTG. Significantly improved *R*^2^ changes in the third step indicated the curvilinear relationship between grief and PTG. Then resource loss was entered in step 4; grief × resources loss interaction was entered in step 5; and grief^2^ × resources loss interaction was entered in step 6. The moderating effect can be verified from step 5 to step 6 by testing the significance of *R*^2^ changes and regression coefficients on interaction terms. Main and interaction effects were centered to minimize multicollinearity. The significance level was set to *α* = 0.05.

Statistical analyses were performed by SPSS version 25 (IBM Corp, 2017), except for the CFA, which was carried out by Mplus Version 7.4 (Muthén and Muthén, 2013).

## Results

### Preliminary Analyses

**Figure 1:**
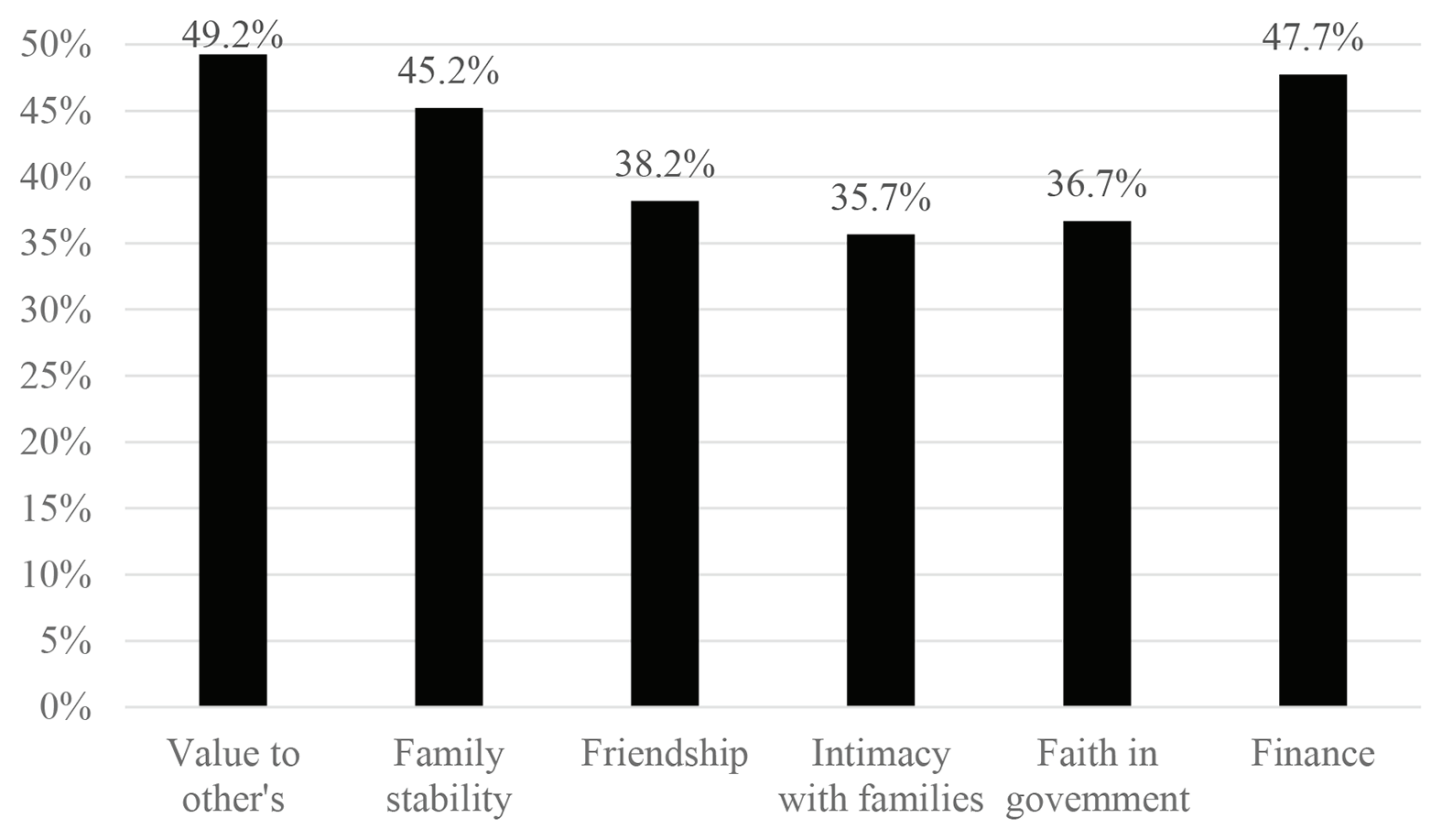
The proportion of each resource loss item shidu parents endorsed “many” and “great.”

Descriptive results of continuous covariates (time since the loss) and psychosocial variables are presented in Table 2. The values of the skewness (0.04–1.23) and kurtosis (−1.18 to 1.52) suggested that none of these variables had unacceptable levels of skew or kurtosis (Tabachnick et al., 2007). The mean total scores of PG-13 and PTGI-CS were (32.97 ± 11.34) and (44.56 ± 15.07). The total scores of interpersonal loss and socio-economic loss were (5.09 ± 3.53) and (2.66 ± 1.94). For each item of resources loss, the proportion of shidu parents who scored 2 or 3 (many or great losses) is shown in Figure 1. Losses in “value to others” (49.2%), “family stability” (45.2%), and “finances” (47.7%) were most endorsed, with almost half of participants feeling that way. Losses in “Intimacy with at least one family member” were the least frequently endorsed in this group, still about a third (35.7%) of shidu parents reported it.

**Table 2:** Descriptive results of continuous covariates and psychosocial variables.

Variable	Mean	*SD*	Range	Skewness	Kurtosis
Time since loss (years)	8.30	6.00	0.5–29.75	1.23	1.51
Grief	32.97	11.34	11–55	0.04	−0.84
PTG	44.56	15.07	18–102	1.04	1.52
Interpersonal loss	5.09	3.53	0–12	0.17	−1.02
Socio-economic loss	2.66	1.94	0–6	0.17	−1.18

PTG, posttraumatic growth.

### Correlations Analyses

The correlations among covariates and main research variables are presented in Table 3. Gender (*r* = 0.381; *p* < 0.001), interpersonal loss (*r* = 0.397; *p* < 0.001), and socio-economic loss (*r* = 0.362; *p* < 0.001) had significant correlations with grief. Only having a grandchild was significantly negatively related to PTG in shidu parents (*r* = −0.201; *p* = 0.004).

**Table 3:** Correlations among covariates and main research variables.

	Grief	PTG	Interpersonal loss	Socio-economic loss
Gender of participants	0.38^**^	0.07	0.05	0.04
Time since loss	−0.10	−0.04	−0.02	−0.05
Cause of death	0.11	0.06	0.05	−0.02
Having a grandchild	−0.02	−0.20^**^	0.01	0.07
Grief	1.00	−0.03	0.40^**^	0.36^**^
PTG	−0.03	1.00	−0.02	0.03

PTG, posttraumatic growth.

** *p* < 0.01.

### Hierarchical Multiple Regression Analyses

The results of HMR analyses (Table 4) did not support our first hypothesis that there was an inverted U-shape model between grief and growth, while the second hypothesis about the moderating role of resource loss was partly supported.

**Table 4:** Hierarchical regression model predicting outcomes of posttraumatic growth (PTG).

Steps and variables	Model 1			Steps and variables	Model 2		
	*β*	*t*	Δ*R*^2^		*β*	*t*	Δ*R*^2^
Step 1			0.06^*^	Step 1			0.06^*^
Gender	2.03	0.92		Gender	2.03	0.92	
Time since loss	−0.25	−1.35		Time since loss	−0.25	−1.35	
Cause of death	1.25	0.58		Cause of death	1.25	0.58	
Having a grandchild	−7.22	−3.07^**^		Having a grandchild	−7.22	−3.07^**^	
Step 2			0.01	Step 2			0.01
grief	−0.12	−1.18		grief	−0.12	−1.18	
Step 3			0.01	Step 3			0.01
grief ^2^	0.01	1.28		grief ^2^	0.01	1.28	
Step 4			<0.01	Step 4			0.01
LIR	0.11	0.34		LSR	0.81	1.36	
Step 5			<0.01	Step 5			0.01
grief × IR	0.02	0.77		grief × SR	0.07	1.30	
Step 6			0.03^*^	Step 6			0.01
grief^2^ × IR	0.01	2.46^*^		grief^2^ × SR	0.01	1.59	
Total *R*	0.32				0.32		
Total *R*^2^	0.10				0.10		
Adjusted *R*^2^	0.06				0.06		

*β*, unstandardized regression coefficients; PTG, posttraumatic growth; IR, interpersonal loss; SR, socio-economic loss.

* *p* < 0.05;

** *p* < 0.01.

Shidu parents’ having a grandchild was significantly negatively associated with PTG (*β* = −7.22, *t* = −3.07, *p* = 0.002, Cohen’s *f*^2^ = 0.048). However, in the third step, the addition of quadratic grief did not show significant *R*^2^ changes (*β* = 0.01, Δ*R*^2^ = 0.01, *p* = 0.202). There was no significant curvilinear relationship between grief and PTG in shidu parents.

When examining the effect of interpersonal loss (Model 1), significant *R*^2^ changes and regression coefficient on interaction terms in step 6 were found (Δ*R*^2^ = 0.03, *β* = 0.01, *p* = 0.015, Cohen’s *f*^2^ = 0.032). There was only a significant interaction effect between grief^2^ and interpersonal loss but not loss of socio-economic resources (Model 2).

**Figure 2:**
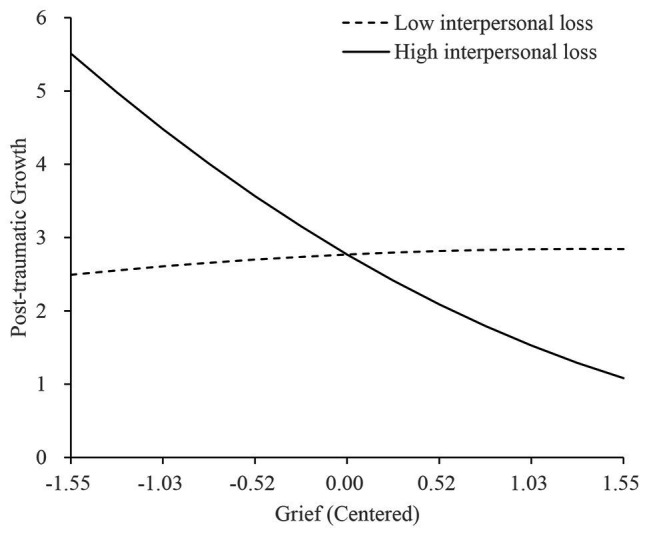
Relationship among grief, PTG, and interpersonal loss among shidu parents.

We explored the moderating effect of interpersonal loss by calculating the relationships between grief^2^ and PTG under low, average, and high levels of interpersonal loss. Under low (1 *SD* below mean) and average levels of interpersonal loss, no significant correlations between grief^2^ and PTG were found (*β* = −0.01, *t* = −0.60, *p* = 0.551; and *β* = 0.01, *t* = 1.49, *p* = 0.139). Under high levels of interpersonal loss (1 *SD* above mean), grief^2^ were significantly related to PTG (*β* = 0.03, *t* = 2.50, *p* = 0.013, Cohen’s *f*^2^ = 0.031). These results indicated a curvilinear relationship between grief and PTG existed at the high interpersonal loss level. For shidu parents with high levels of interpersonal loss, as the severity of grief symptoms increased, their PTG levels decreased (Figure 2).

## Discussion

The current study examined the relationship between grief and PTG, and the moderating role of resources-loss among Chinese bereaved parents who lost their only child. The results of the current study did not support an inverted U-shape relationship between grief and PTG in shidu parents. When testing the moderating effects of two kinds of resource loss, we found that under the high levels of interpersonal loss, shidu parents’ PTG decreased as the severity of grief increased. However, the socio-economic loss did not show a significant moderating effect.

Shidu parents’ having a grandchild was significantly negatively associated with PTG. Chinese culture regarding filial piety and continuation of bloodlines may be the main reason. The death of the only child would challenge shidu parents’ value of continuation of the family line. In this situation, having a grandchild may be a comfort and could alleviate some pain for these shidu parents. Death may challenge their world belief to a lesser degree when they still have an offspring to continue their bloodlines. Therefore, it might result in less effort to make meaning of death and less reported growth (Calhoun et al., 2010).

There was no significant curvilinear relationship between grief and PTG in shidu parents. This finding was surprising given the support from previous studies (Currier et al., 2012; Yilmaz and Zara, 2016). One of the reasons to account for this difference might be related to sample characteristics. Participants in the above two studies were bereaved adults who experienced all kinds of bereavement, not confined to a child-loss. However, in our study, the participants were bereaved parents who lost their only child and their average age was 60.92 years. Besides, their average time since the loss was 8.30 years, but which was limited to 2 years in the other two studies. Time since the loss seems to be an important factor associated with PTG (Waugh et al., 2018). A study on bereaved youth also did not find this curve relationship (Salloum et al., 2019). These results suggested that the patterns of the relationship between grief and growth may vary in different samples. The curvilinear relationship between grief and PTG in adults indicated that too low levels of grief may be not enough to challenge ones’ assumptive world beliefs, while too high levels of grief probably will overwhelm an individual’s capacity for growth (Currier et al., 2012; Eisma et al., 2019). However, in addition to the grief levels, the levels of disruption of core beliefs and the extent to which bereaved people have reconstructed their beliefs about the world must be taken into account (Calhoun et al., 2010). Child-loss is considered as the most severe kind of bereavement, and it violates the belief that children should outlive their parents (Zetumer et al., 2015). It is often perceived as unnatural and unjust (Hibberd et al., 2010). To most Chinese parents, children mean their hope, emotional support, the continuation of bloodlines, and main caregivers after getting old. Losing the only child may mean losing all of these. Therefore, understanding the only child’s death, rebuilding a functional worldview, and achieving PTG may be more difficult and complicated for shidu parents. Due to the lack of qualitative and follow-up information, the process of grief and PTG, and how reconstructed beliefs work among shidu parents are not very clear so far. Therefore, qualitative research and longitudinal studies are necessary to further explore the relationship between grief and PTG among shidu parents.

Only interpersonal loss but not socio-economic loss showed a significant moderating effect between grief and PTG. The value of Cohen’s *f*^2^ of 0.032 indicated that the moderating effect was smaller than medium effect sizes but not so small as to be trivial (Cohen, 1988). The conclusion is similar to previous findings that the interpersonal loss had a significant effect after trauma (Banou et al., 2009; Hall et al., 2015).

When exploring the moderating effect of interpersonal loss, we found that more severe grief symptoms were related to lower PTG when the interpersonal loss is higher. Some reasons may account for this phenomenon. Severe interpersonal loss may lead to a lack of adequate social support for the bereaved to adapt to loss. Social support may alleviate the negative effects of the loss and facilitate effective coping, which is considered an important factor in facilitating PTG (Howard Sharp et al., 2018; Oginska-Bulik, 2018). For shidu parents with higher levels of grief, being “stuck” in grief, such as longing for and preoccupation with their deceased child, leaves them limited time and energy to search for new meanings and direction in life. When it is coupled with severe interpersonal loss, shidu parents may use out all the resources and lack sufficient interpersonal support to struggle with the grief and loss. Therefore, it is hard for shidu parents with high levels of grief to achieve growth under severe interpersonal loss. Additionally, higher resources loss and grief are related to lower self-efficacy (Smith et al., 2015; Witting et al., 2016; Edwards et al., 2018), while self-efficacy is positively related to PTG (Shakespeare-Finch et al., 2014). Consequently, under high levels of interpersonal loss, shidu parents’ higher grief is related to lower PTG.

Socio-economic loss, including loss of faith in government and financial loss, did not play a significant role in the relationship between grief and PTG. Because the only child’s loss is related to the previous national policy, shidu parents may have conflicted feelings toward the government. On the one hand, they may experience certain levels of resentment and loss of faith in the government. On the other hand, they still rely on the government and realize that the government provides support for them (Zhang and Jia, 2018). Besides, a monthly subsidy from the government can make up for their financial loss to a certain extent. Shidu parents could be supported by the government, but the significant socio-economic loss has been reported nonetheless. The government support and their reliance on government may mitigate the impact of socio-economic loss. These might explain why economic loss did not play a significant role. Additionally, perhaps the more significant impact of interpersonal loss on shidu parents may be another reason. Self-identity plays a core role in bereavement adjustment (Maccallum and Bryant, 2013). Chinese shidu parents show a typical merged self-identity and a weak independent self-identity (Yu, 2018). They might very strongly define themselves through relationships and are affected by others. Therefore, the interpersonal loss would have a stronger effect on grief and PTG.

### Implications

The findings of the current study expand the bereavement research under Chinese cultural contexts and provide some guidance for clinical treatment in the area of grief counseling. First, shidu parents’ pattern of grief and PTG is different from that in bereaved adults in Western culture (Currier et al., 2012; Eisma et al., 2019). This result implies that there may be some unique characteristics among shidu parents. Besides, some researchers have proposed a two-component model of PTG, indicating that PTG has a functional side and meanwhile may also contain illusory, self-deceptive components, thereby it may not always be beneficial for adjustment (Zoellner and Maercker, 2006). The role of PTG in bereavement is still open to discussion. More studies, especially longitudinal studies about the adaptive significance of PTG and its related factors in shidu parents are highly needed. Secondly, the present study highlighted the moderating role of interpersonal loss between grief and PTG that might provide some information for interventions. It seems essential for therapists to raise awareness of the possibility of PTG and have an understanding of what factors may be related to PTG (Zoellner and Maercker, 2006). The current study results indicated that high levels of grief coupled with high levels of interpersonal loss may interfere with shidu parents’ ability to achieve PTG. Therefore, evaluating degrees of interpersonal loss seems to be an important part of the treatment process when working with shidu parents. For people who experienced higher interpersonal loss, professional grief treatments that not only focus on the loss but also help the bereaved strengthen interpersonal relationships can be considered, such as complicated grief treatment (CGT; Shear et al., 2005, 2016). CGT has an emphasis on helping bereaved people restore their connections to others in their life. When implementing CGT with shidu parents, this theme deserves close attention to help them maintain existing or develop new interpersonal relationships. Additionally, outside of the clinical context, shidu parents with severe interpersonal loss and grief may benefit from social support. Previous studies suggest that different kinds of social support may have different impacts on different bereaved people (Li and Chen, 2016; Howard Sharp et al., 2018). Future research is encouraged to explore what types of social support (e.g., emotional support or practical support) or which sources of social support (i.e., family, friends, and significant others) may be most helpful for shidu parents.

### Limitations

The study has several limitations. First, many shidu parents with severe grief symptoms or avoidance of social contact may be unwilling to participate in the present study. Therefore, the current study was based on a convenience sampling method, and only 199 participants were included in the analyses. Though this was a useful strategy in recruiting this hard-to-reach population, which may limit the generalizability of the findings. The result of *post hoc* power analysis (with the estimation based on *α* = 0.05 and effect size = 0.032, power = 0.713) indicated the statistical power to identify a significant moderating effect was limited due to the relatively small sample size. Besides, most of the participants in the current study lost an adult child. Therefore, cautious interpretation is required when extending the results to the whole shidu parents. Future research using a large, representative sample is encouraged to validate these findings. Second, we only used a questionnaire method to explore bereavement outcomes. The revised version of PTGI for Chinese shidu parents was used for the first time. Besides, perhaps because the socio-economic loss subscale only has two items, the alpha coefficient of it was relatively low. With the limitation of research methods, we failed to learn about deeper information about the relationship between grief and PTG, and the effects of resources loss. Future studies combining questionnaire and interview methods might provide us with a more comprehensive understanding. Third, this is a cross-sectional study that does not allow causal conclusions about grief, PTG, and resource loss. Consequently, longitudinal studies may be needed to further expand on these findings.

## Conclusion

The current study focused on a special group of bereaved parents: parents who lost their only child. Results did not reveal a significant curvilinear relationship between grief and PTG in shidu parents. Under severe interpersonal loss, shidu parents’ high levels of grief symptoms may interfere with the progress of PTG. Therefore, the evaluation of degrees of interpersonal loss is necessary and meaningful when working with shidu parents. These findings not only enrich the bereavement research in eastern culture, but also provide a potential benefit to therapists and social workers to develop a specific treatment to help shidu parents from a positive perspective.

## Data Availability Statement

The datasets generated for this study are available on request to the corresponding author.

## Ethics Statement

The studies involving human participants were reviewed and approved by Ethics Committee of Beijing Normal University. The patients/participants provided their written informed consent to participate in this study.

## Author Contributions

XX collected data for the study and drafted the initial manuscript. JW collected and analyzed the data and reviewed and edited the manuscript. NZ, GS, and RT collected data for the study and reviewed the manuscript. JW was responsible for project management and supervision. NS contributed substantially to the manuscript revision and editing. All authors contributed to the article and approved the submitted version.

### Conflict of Interest

The authors declare that the research was conducted in the absence of any commercial or financial relationships that could be construed as a potential conflict of interest.
